# Supplementary material for: HPV Vaccine Hesitancy and Influencing Factors among University Students in China: A Cross-Sectional Survey Based on the 3Cs Model
Source: Int J Environ Res Public Health. 2022 Oct 28;19(21):14025. doi: 10.3390/ijerph192114025 (PMC9657119; doi:10.3390/ijerph192114025)
Supplement: Supplementary file 1 [file ijerph-19-14025-s001.zip › ijerph-1964583-supplementary.pdf]

International Journal of Environmental Research and Public Health

MDPI

Supplementary materials:

Table S1. Multinomial logistic regression to identify factors associated with HPV vaccine hesitancy (full model)

	Factors		Hesitancy vs. No hesitancy				Refusal vs. No hesitancy			
			OR	*p*-Value	95% CI		OR	*p*-Value	95% CI	
Complacency	The severity of being infected by HPV	(Rf: High)								
		Low	4.007	**0.003**	1.584	10.138	3.362	0.051	0.996	10.758
		Neutral	1.349	0.369	0.702	2.593	1.097	0.821	0.491	2.453
	The probability of being infected by HPV	(Rf: High)								
		Low	0.537	0.437	0.112	2.577	0.833	0.766	0.25	2.779
		Neutral	0.864	0.868	0.154	4.833	0.946	0.920	0.319	2.802
		(Ref: Very fear)								
	The fear of being infected by HPV	Moderate	0.568	0.460	0.127	2.547	1.821	0.298	0.589	5.628
		Mild	0.436	0.278	0.097	1.954	1.393	0.468	0.569	3.410
	The necessity of HPV vaccination	(Ref : Unnecessary)								
		Neutral	0.143	0.180	0.008	2.458	0.143	0.180	0.008	2.458
		Necessary	0.198	0.275	0.011	3.641	0.198	0.275	0.011	3.641
Confidence	The negative information about vaccines	(Ref: No)								
		Yes	1.026	0.933	0.565	1.862	0.862	0.722	0.380	1.953
		(Re: Distrust)								
	The safety of domestic vaccines	Neutral	1.718	0.614	0.210	14.076	1.361	0.806	0.117	15.876
		Trust	1.566	0.683	0.182	13.515	1.342	0.810	0.121	14.841
		(Re: Distrust)	.	.	.	.	.	.	.	.
	The safety of vaccines abroad	Neutral	2.976	0.687	0.015	60.831	0.327	0.638	0.003	34.456
		Trust	2.787	0.707	0.013	57.463	0.404	0.703	0.004	42.466
	The efficacy of vaccines	(Re: Distrust)								
		Total trust	0.084	**0.006**	0.014	0.494	0.898	0.880	0.220	3.669
		Neutral	0.112	**0.009**	0.021	0.583	0.854	0.746	0.329	2.214
	The vaccine-related advice provided by medical staffs is reliable	(Re: Distrust)								
		Neutral	3.488	0.831	0.3616	3.364	1.846	0.84	0.005	71.85
		Trust	3.549	0.828	0.3749	3.359	1.337	0.923	0.004	49.60
Convenience	Price prevents from vaccinating against HPV	(Ref: No)								
		Yes	0.128	**<0.001**	0.055	0.299	0.540	0.223	0.201	1.455
	Time/distance prevents from vaccinating	(Ref: No)								
		Yes	15.366	**<0.001**	6.702	35.229	0.333	0.087	0.094	1.174
Other	City	(Ref :Nanning)								
		Shanghai	0.816	0.566	0.408	1.632	0.861	0.809	0.255	2.903
		Wuhan	0.815	0.497	0.452	1.471	1.161	0.771	0.425	3.171
		Guangzhou	0.912	0.778	0.480	1.732	0.882	0.849	0.244	3.195
		(Ref: >26)								
	Age (years)	20-26	0.479	0.259	0.133	1.719	0.251	0.155	0.037	1.686
		<20	0.595	0.397	0.179	1.979	0.264	0.138	0.045	1.537
	Education Level	(Ref: Postgraduate)								
		Junior college	4.109	**0.010**	1.396	12.095	0.758	0.718	0.169	3.402

	Undergraduate	3.601	**0.004**	1.514	8.561	1.140	0.810	0.393	3.311
Family history of cancer	(Ref: No)								
	Yes	0.378	**0.049**	0.137	1.116	4.069	0.008	1.434	11.542
HPV understanding	(Ref: Low scores (0-3))								
	High scores (7-9)	0.140	**0.035**	0.029	0.837	2.166	0.077	0.921	5.095
	Moderate scores (4-6)	0.443	**0.017**	0.243	0.838	1.047	0.913	0.464	2.362
Major	(Ref: Medicine)								
	Science and technology or agriculture	0.778	0.501	0.375	1.615	0.952	0.942	0.254	3.564
	Social sciences	1.091	0.731	0.664	1.792	1.055	0.907	0.429	2.591
Residence	(Ref: Urban)								
	Rural	1.094	0.666	0.727	1.647	0.972	0.942	0.456	2.073
Medical insurance	(Ref: No)								
	Yes	0.811	1.098	0.511	2.357	1.613	0.503	0.398	6.533
Living expense (CNY)	(Ref: >3000)								
	<1000	6.472	0.077	0.819	5.149	0.683	0.687	0.107	4.367
	1001-2000	4.657	0.133	0.625	3.668	0.436	0.321	0.085	2.247
	2001-3000	5.875	0.086	0.776	4.465	0.359	0.247	0.063	2.037
Have been vaccinated at own expense in the past 5 years	(Ref: No)								
	Yes	0.715	0.201	0.427	1.197	0.988	0.978	0.407	2.397

Table S2 Model Fitting Information

Model	Model Fitting Criteria	Likelihood Ratio Tests	*p*-Value
	-2 Log Likelihood	Chi-square	
Intercept Only	1067.713		
Final	882.96	184.753	<0.001

Table S3 Likelihood Ratio Tests

Factors	Model Fitting Criteria	Likelihood Ratio Tests	Df	*p*-Value
	-2 Log Likelihood of Reduced Model	Chi-square		
Education Level	896.440	13.479	3	0.009
Family history of cancer	901.624	18.664	2	<0.001
Knowledge of HPV and HPV vaccine	927.877	44.917	3	<0.001
The efficacy of vaccines	916.846	33.885	3	<0.001
The severity of being infected by HPV	900.229	17.268	3	0.002
Price prevents from vaccinating against HPV	484.851	14.633	2	0.001
Time/distance prevents you from vaccinating against HPV	460.395	10.178	2	0.006
